# Book Notices

**Published:** 1868-05

**Authors:** 


### Book Notices.

Atlas of Venereal Diseases. By A. CULLERIER, Surgeon to the Hospital Du Mida; Member of the Surgical Society of Paris; etc., etc., etc. Translated from the French, with notes and additions, by FREEMAN J. BUMSTEAD, M.D., Prof. of Venereal Diseases in the College of Physicians and Surgeons of New York. With about One Hundred and Fifty Beautifully Colored Figures on Twenty-Six Plates. Philadelphia: HENRY C. LEA. 1868. To be completed in five parts, $3 each.

This is one of the most elegantly published and valuable works that ha been reprinted and edited in this country, relating to the loathsome, though important, class of venereal diseases. The author and editor are alike men of experience and eminent ability; and we freely commend this product of their labors to the general patronage of the profession. Parts I and II are already in our bookstores.

The Diagnosis, Pathology, and Treatment of Diseases of Women, Including the Diagnosis of Pregnancy. By GRAILY HEWITT, M.D., London, F.R.C.P., Prof. Midwifery and Diseases of Women, University College, and Obstetric Physician to the Hospital, etc., etc. First American, from the Second London Edition, Revised and Enlarged. With 116 Illustrations. Philadelphia: LINDSAY & BLAKISTON. 1868.

This is a handsome octavo volume of 707 pages. It includes a consideration of all the diseases and accidents usually regarded as peculiar to the female sex, and is well adapted both as a text-book for students and as a work of reference for the practitioner. We have neither time nor space to speak of its contents in detail.

---

Electro-Physiology and Therapeutics: Being a Study of the Electrical and other Physical Phenomena of the Muscular and other Systems during Health and Disease, including the Phenomenon of Electrical Fishes. By CHARLES E. MORGAN, A.B., M.D. New York: WM. WOOD & CO. 1868.

This is certainly one of the most important works of a scientific character, that has been issued from the American Press. It contains 714 pages, closely printed; and presents the reader with a complete review of all that is known in relation to electricity and its connection with the phenomena of living matter in health and disease. It merits a place in the library of every physician and cultivator of the natural sciences.

---

A Treatise on General Pathology, and its Relations to Practical Medicine. By CHARLES L. CARTER, M.D., Honorary Member of the St. Louis Medical Society; lately Surgeon in U.S. Army. St. Louis: GEORGE KNAPP & CO. 1867.

This is an octavo volume of 149 pages, on fair type and paper. It embraces a discussion of the topics usually included under the head of general pathology. The special merits of the work we shall examine more carefully hereafter.

---

Lectures on the Causes, Pathology, and Treatment of Joint Diseases. Delivered at the McGill University Medical College, Montreal, Canada. By LOUIS BAUER, M.D., M.R.C.S., Eng., Prof. of Anatomy and Clinical Surgery, etc., etc., etc. Reprinted from the Canada Medical Journal. New York: WM. WOOD & CO., 61 Walker Street. 1868. Pp. 96.

This is an octavo volume, well illustrated with cuts. It is just what its title indicates, namely, a monograph on the causes, pathology, and treatment of diseases of the joints. The author is an independent thinker, thoroughly conversant with his subject, theoretically and practically. Hence, his work can be read with pleasure and profit both by students and practitioners.

---

Annual Abstract of Therapeutics, Materia Medica, Pharmacy, and Toxicology, for 1867; followed by an Original Memoir On Gout, Gravel, and Urinary Calculi, by A. BOUCHARDAT, Prof. of Hygiene to the Faculty of Medicine, Paris; etc., etc., etc. Translated and Edited by M. J. DE ROSSET, M.D., Adjunct to the Professor of Chemistry in the University of Maryland, etc., etc. Philadelphia: LINDSAY & BLAKISTON. 1868.

This is a duodecimo volume of 314 pages, neatly bound. Its contents are well indicated by the above title. It will be found very useful to both physicians and druggists.

---

Felix Von Niemeyer's Clinical Lectures on Pulmonary Phthisis. Translated, by permission of the Author, from the Second German Edition, by J. L. PARKE. New York: MOORHEAD, SIMPSON, & BOND. 1868.

For sale by the Western News Co., Chicago.

